# Mechanism of KLF9 in airway inflammation in chronic obstructive pulmonary

**DOI:** 10.1002/iid3.1043

**Published:** 2023-10-12

**Authors:** Peijie Gu, Zhen Wang, Xin Yu, Nan Wu, Liang Wu, Yihang Li, Xiaodong Hu

**Affiliations:** ^1^ Department of Pulmonary and Critical Care Medicine Jiangyin Hospital of Traditional Chinese Medicine Jiangyin City China

**Keywords:** airway inflammation, bronchoalveolar lavage fluid, chronic obstructive pulmonary disease, cigarette smoke extract, KLF9, miR‐494‐3p, NLRP3, PTEN

## Abstract

**Background:**

Chronic obstructive pulmonary disease (COPD) is an airway‐associated lung disorder, resulting in airway inflammation. This article aimed to explore the role of the krüppel‐like factor 9 (KLF9)/microRNA (miR)‐494‐3p/phosphatase and tensin homolog (PTEN) axis in airway inflammation and pave a theoretical foundation for the treatment of COPD.

**Methods:**

The COPD mouse model was established by exposure to cigarette smoke, followed by measurements of total cells, neutrophils, macrophages, and hematoxylin and eosin staining. The COPD cell model was established on human lung epithelial cells BEAS‐2B using cigarette smoke extract. Cell viability was assessed by cell counting kit‐8 assay. miR‐494‐3p, KLF9, PTEN, and NLR family, pyrin domain containing 3 (NLRP3) levels in tissues and cells were measured by quantitative real‐time polymerase chain reaction or Western blot assay. Inflammatory factors (TNF‐α/IL‐6/IL‐8/IFN‐γ) were measured by enzyme‐linked immunosorbent assay. Interactions among KLF9, miR‐494‐3p, and PTEN 3′UTR were verified by chromatin immunoprecipitation and dual‐luciferase assays.

**Results:**

KLF9 was upregulated in lung tissues of COPD mice. Inhibition of KLF9 alleviated airway inflammation, reduced intrapulmonary inflammatory cell infiltration, and repressed NLRP3 expression. KLF9 bound to the miR‐494‐3p promoter and increased miR‐494‐3p expression, and miR‐494‐3p negatively regulated PTEN expression. miR‐494‐3p overexpression or Nigericin treatment reversed KLF9 knockdown‐driven repression of NLRP3 inflammasome and inflammation.

**Conclusion:**

KLF9 bound to the miR‐494‐3p promoter and repressed PTEN expression, thereby facilitating NLRP3 inflammasome‐mediated inflammation.

## Graphical abstract

Chronic obstructive pulmonary disease (COPD) is an airway‐associated lung disorder, resulting in airway inflammation. This article aimed to explore the role of the krüppel‐like factor 9/microRNA‐494‐3p/phosphatase and tensin homolog axis in airway inflammation and pave a theoretical foundation for the treatment of COPD.

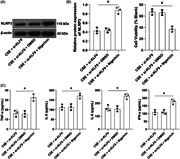

## 1 INTRODUCTION

Chronic obstructive pulmonary disease (COPD) is defined as a prevalent and curable disorder featuring extensive respiratory symptoms and progressive airflow obstruction.^1^ The occurrence of COPD is associated with frequent exposure to inhaled noxious particles, prominently cigarette smoke.^2^ Airway inflammation is a consistent pathological trait of COPD and results from interplay of inflammatory cells (e.g., macrophages, neutrophils, and T lymphocytes), structure cells, and oxidative stress; COPD‐accelerated aging in the lung also promotes the release of inflammatory mediators and proteins.^3^ Nevertheless, the current first‐line treatment aims at alleviation of bronchoconstriction by means of bronchodilators, instead of anti‐inflammation.^4^ Against this backdrop, the identification of relevant biomarkers helps develop personalized approaches to quench airway inflammation.

The inflammatory responses in the airway are accompanied by the production of inflammasome, chemokines, cytokines, and pro‐inflammatory transcription factors. NLR family, pyrin domain containing 3 (NLRP3) inflammasome is a multimeric complex consisting of three parts NLRP3 receptor protein, pro‐caspase‐1, and apoptosis speck‐like protein and is implicated in airway inflammation in both COPD and asthma.^5^ In addition, the effectors of NLRP3 inflammasome, such as interleukin (IL)‐1β, NLRP3, and caspase‐1 are found to be elevated in plasma of COPD patients and IL‐1β combined with white blood cells, and fibrinogen shows predictive value in 89% of cases.^6^ On a separate note, krüppel‐like factors (KLFs) represent a set of zinc finger domain‐containing transcription factors and are crucial players in various pulmonary disorders, such as pulmonary arterial hypertension, pulmonary hypoplasia, and lung fibrosis.^7^, ^8^, ^9^ Especially, KLF9 has been reported to be elevated in COPD lungs, suggesting its involvement in COPD pathogenesis.^10^ Currently, there is no functional study of KLF9 in COPD‐induced airway inflammation.

MicroRNAs (miRNAs) are small noncoding RNA molecules with 19–25 nucleotides with an ability to negatively regulate message RNAs (mRNAs) through recognition of 3′ untranslated region (UTR).^11^ Cigarette smoke and air pollutants can induce alteration of miRNA expression and miRNAs further regulate pathological traits of COPD, such as oxidative stress, apoptosis, and inflammatory responses.^12^ Of note, miR‐494‐3p is a well‐documented miRNA regulating tumorigenesis, angiogenesis, cell death, inflammation, and immunity.^13^, ^14^, ^15^, ^16^ Silencing of miR‐494‐3p is shown to moderate senescence and inflammation in COPD.^17^ Significantly, the JASPAR database uncovered the binding of KLF9 to the miR‐494‐3p promoter, suggesting their interaction in COPD. The phosphatase and tensin homolog (PTEN) is a genetic regulator of various metabolic changes, including glycolysis, lipid metabolism, and mitochondrial metabolism.^18^ PTEN also emerges as a potential target for various chronic lung diseases, including COPD, asthma, pulmonary hypertension, and acute lung injury, and regulates physiological activities ranging from inflammation responses, apoptosis to proliferation.^19^ Additionally, the binding of miR‐494‐3p to PTEN 3′UTR sequence was predicted and verified by databases and the dual‐luciferase assay, suggesting miR‐494‐3p‐mediated negative regulation of PTEN in COPD.

Given the above associations, we speculated that the KLF9/miR‐494‐3p/PTEN axis plays a role in COPD‐induced airway inflammation. The objective of our study is to identify a novel molecular route for airway inflammation and provide potential therapeutic targets for COPD.

## 2 MATERIALS AND METHODS

### 2.1 Experimental animals and model establishment

The scheme of animal experiments was ratified by the Animal Care and Use Committee of Jiangyin Hospital of Traditional Chinese Medicine and abided by the Guide for the Care and Use of Laboratory Animals.^20^ A total of 32 male C57BL6J mice aged 6‐8 weeks were allocated to different groups with 8 mice per group. Mice in the control group were exposed to the air, while mice in the experimental groups were exposed to cigarette smoke. Mice were placed in a toughened glass case and stimulated by smoke from 20 cigarettes (10 mg tar and 0.8 mg nicotine per cigarette) for 2 h twice a day, 7 days a week, which lasted for 6 months. Control mice were kept in a similar environment with experimental mice but without stimulation of smoke.

Short hairpin RNA (shRNA) targeting KLF9 was constructed and packaged by lentivirus. At the first, second, third week of COPD modeling, mice were injected with lentivirus vectors (Shanghai GenePharma Co., Ltd.) via the caudal vein with virus titer of 5 × 10^7^ TU/mL and injection volume of 2 × 10^7^ TU.

### 2.2 Collection of bronchoalveolar lavage fluid

After anesthesia and tracheal intubation, mice underwent intrapulmonary injection with cold Dulbecco's modified Eagle medium (DMEM) to obtain bronchoalveolar lavage fluid (BALF). The total number of cells was measured using a Countess II automatic cell counter. After spin centrifugation, the obtained cells were fixed and examined by modified Diff‐Quick staining, with the use of a cytospin slide to measure the number of differentiated cells. After obtaining BALF, mice were euthanatized with pentobarbital (200 mg/kg), and the lungs were taken, with the left lung used for histological staining and the right lung prepared into homogenate for the subsequent assays.

### 2.3 Hematoxylin and eosin staining

The left lung was subjected to injection with 1 mL of 10% formalin through the trachea and 24 h immersion in fixing solution. After paraffin embedding, tissue blocks were sliced into 6 μm sections, followed by hematoxylin and eosin (H&E) staining. The stained tissues were observed by optical microscopy.

### 2.4 Preparation of cigarette smoke extract

The preparation of cigarette smoke extract (CSE) was conducted according to the previous report.^21^ The smoke from two cigarettes was dissolved in 10 mL serum‐free DMEM, forming CSE solution with a 7.4 pH value. Next, CSE solution was filtered through a 0.22‐μm pore filter to wipe out insoluble particles and was standardized by absorbance measurement at 320 and 540 nm, which was defined as 100% CSE. Following DMEM‐dependent dilution to 5% concentration, CSE diluent was used within 30 min.

### 2.5 Cell culture and treatment

Human lung epithelial cell lineage BEAS‐2B (with the origin of sampling site: bronchus; epithelium) was brought from ATCC corporation and underwent convention culture (DMEM, 10% fetal bovine serum [FBS], 100 U/mL penicillin, 100 μg/mL streptomycin). After that, cells were treated with 5% CSE for 24 h to establish the COPD cell model.

Small interfering RNAs (siRNA) targeting KLF9 (si‐KLF9‐1 and si‐KLF9‐2), miR‐494‐3p mimic (mimic‐494), and the corresponding controls (si‐NC and mimic‐NC) were designed by Shanghai Genechem Co., Ltd. Seeding into the six‐well plate, reaching 70%–80% confluence, cells were transfected with the above siRNAs and mimics. With the guidance of the Lipofectamine 3000 protocol (Thermo Fisher Scientific, Inc.), 2500 ng siRNA or mimic, 7.5 µL Lipo3000, and 5 µL p3000 were dissolved into 250 µL opti‐MEM and merged for 30 min in each well. Subsequently, the compound was transferred to the six‐well plate, followed by the addition of opti‐MEM medium to 2 mL volume in each well. After 4–6 h of transfection, the culture medium was replaced by DMEM containing 10% FBS. After 48 h of transfection, CSE was incorporated according to the experimental grouping. NLRP3 activator Nigericin (MedChemExpress, LLC) was used to treat cells at 10 µM concentration at the same time of CSE treatment, with dimethylsulfoxide serving as the control.

### 2.6 Cell counting kit‐8 assay

In accordance with the instruction (Yeasen, Shanghai, China), cells underwent seeding into the 96‐well plate at a density of 2.0 × 10^4^ cells/well and later different treatments in the incubator. After 48 h incubation, 10 μL of cell counting kit‐8 solution was incorporated into each well, followed by another 2 h incubation at 37°C. A microplate reader was used to measure absorbance at 450 nm wavelength.

### 2.7 Quantitative real‐time polymerase chain reaction

The extraction of the total RNA from lung tissues and cells was conducted by means of the TRIzol reagent (Invitrogen) and later conversion into the complementary DNA was realized using the FastKing cDNA first‐strand synthesis kit (Tiangen). Subsequently, quantitative real‐time polymerase chain reaction was implemented with the help of AceQ Universal SYBR qPCR Master Mix (Vazyme) and ABI 7500 system (Applied Biosystems). With glyceraldehyde‐3‐phosphate dehydrogenase serving as the internal genes for mRNAs and U6 as the internal reference for miRNA,^22^ the relative expression amount was calculated based on the $2^{-{\mathrm{\Delta \Delta C}}_{t}}$ method. Used primers are exhibited in Table 1.

**Table 1:** PCR primer sequence information.

Gene	Sequence (5′‐3′)
hsa‐KLF9	F: GCCGCCTACATGGACTTCG
	R: GGATGGGTCGGTACTTGTTCA
hsa‐miR‐494‐3p	F: GCCGAGTGAAACATACACGG
	R: CTCAACTGGTGTCGTGGA
hsa‐PTEN	F: TTTGAAGACCATAACCCACCAC
	R: ATTACACCAGTTCGTCCCTTTC
hsa‐GAPDH	F: GGAGCGAGATCCCTCCAAAAT
	R: GGCTGTTGTCATACTTCTCATGG
hsa‐U6	F: GTGCTCGCTTCGGCAGCA
	R: AAAATATGGAACGCTTCA
mmu‐KLF9	F: TGGAGAGTCCCGATGAGGATA
	R: GAGGCGTGTTTCCCCTTCG
mmu‐miR‐494‐3p	F: GCCGAGTGAAACATACACGG
	R: CTCAACTGGTGTCGTGGA
mmu‐PTEN	F: TGGATTCGACTTAGACTTGACCT
	R: GCGGTGTCATAATGTCTCTCAG
mmu‐GAPDH	F: AGGTCGGTGTGAACGGATTTG
	R: TGTAGACCATGTAGTTGAGGTCA
mmu‐U6	F: GTGCTCGCTTCGGCAGCA
	R: AAAAATATGGAACGCTTCA
hsa‐miR‐494‐3p promoter	F: GTGCCACACAGGGGCCTGGGC
	R: GGAGCTTTCCTGACGGTGGA

Abbreviations: GAPDH, glyceraldehyde‐3‐phosphate dehydrogenase; PCR, polymerase chain reaction.

### 2.8 Western blot assay

Lung tissues and cells were lysed with the addition of radioimmunoprecipitation assay and protease inhibitor (Beyotime Institute of Biotechnology). The protein concentration was quantified with the use of the bicinchoninic acid kit (Thermo Fisher Scientific, Inc.). Through 10% sodium dodecyl sulfate‐polyacrylamide gel electrophoresis, protein samples were separated and transferred onto polyvinylidene fluoride membranes (Millipore Corp.). Followingly, the membranes were blockaded with Tris Buffered Saline Tween (TBST) containing 5% nonfat milk at environment temperature for 2 h and then incubated with antibodies against KLF9 (1:1000, 701888, Invitrogen), PTEN (1:1000, ab170941, Abcam), NLRP3 (1:1000, ab263899, Abcam), β‐actin (1:1000, ab8227, Abcam) at 4°C overnight. After three washes with TBST for 15 min for each time, the membrane incubation was conducted on horseradish peroxidase‐conjugated secondary antibody (1:2000, ab6721, Abcam) at 37°C for 2 h. The immunoreactive images were captured using the enhanced chemiluminescence kit (Sigma‐Aldrich) and determined by ChemiDoc™ imaging system (Bio‐Rad), with β‐actin used for internal reference.

### 2.9 Enzyme‐linked immunosorbent assay

The levels of tumor necrosis factor (TNF)‐α, IL‐6, IL‐8, interferon‐gamma (IFN‐γ) in BALF or cells were determined by enzyme‐linked immunosorbent assay (ELISA) kits. The used assay kits were as follows: mouse TNF‐α ELISA kit (MBS175787, MyBioSource), human TNF‐α ELISA kit (MBS8420080, MyBioSource), mouse IL‐6 ELISA kit (MBS2023471, MyBioSource), human IL‐6 ELISA kit (MBS2021124, MyBioSource), mouse IL‐8 ELISA (MBS7606860, MyBioSource), human IL‐8 ELISA kit (MBS2019724, MyBioSource), mouse IFN‐γ ELISA kit (MBS825085, MyBioSource), and human IFN‐γ ELISA kit (MBS8413326, MyBioSource).

### 2.10 Bioinformatics

The binding site of KLF9 and the miR‐494‐3p promoter was predicted with the help of the JASPAR database (https://jaspar.genereg.net/).^23^ The downstream target genes of miR‐494‐3p were predicted online with the help of TargetScan database (http://www.targetscan.org/),^24^ miRWalk (http://mirwalk.umm.uni-heidelberg.de/),^25^ and miRTarBase database (http://mirtarbase.cuhk.edu.cn/php/index.php),^26^ from which intersections were identified.

### 2.11 Chromatin immunoprecipitation

Chromatin immunoprecipitation (ChIP) assay was conducted using the ChIP kit (ab500, Abcam). First, equal amounts of cells were treated with 1% methanol for 10 min crosslink, washed with phosphate buffer saline, and resuspended in ChIP nuclear lysis buffer. Next, the crosslinked DNA was ultrasonically processed to generate chromatin fragments. After the lysates were clarified by centrifugation, the supernatant was incubated with the antibody against KLF9 (1:1000, 701888, Invitrogen) or immunoglobulin G (IgG; 1:100, ab6757, Abcam) at 4°C overnight. Subsequently, with an equal amount of pre‐clarified chromatins used as input, chromatins were incubated with fully resuspended protein A/G beads. After washing beads, immunocomplex was eluted by heating at 62°C and shaking. The crosslink of protein‐DNA complex was reversed after 2 h incubation. After purification, immunoprecipitated DNA was analyzed by PCR, with information of PCR primers shown in Table 1.

### 2.12 Dual‐luciferase reporter assay

The miR‐494‐3p promoter region sequence containing the binding site with KLF9 and the mutant sequence fragments were inserted into luciferase plasmids to construct miR‐494‐wild type (WT) and miR‐494‐mutant type (MUT), followed by cotransfection with KLF9 overexpression vector (oe‐KLF9) or the corresponding control (oe‐NC) into BEAS‐2B cells. Likewise, PTEN 3′UTR sequence containing the binding site with miR‐494‐3p and the mutant sequence fragments were inserted into luciferase plasmids to construct PTEN‐WT and PTEN‐MUT, followed by cotransfection with miR‐494‐3p mimic or mimic‐NC into BEAS‐2B cells. After 48 h, cells were collected and tested by the dual‐luciferase reporter assay system (Promega). The relative luciferase activity of each sample was analyzed as the activity of firefly relative to Renilla. The above vectors were designed and constructed by GenePharma Corporation.

### 2.13 Statistical analysis

Statistical analysis and graphing of all data were conducted with the help of SPSS21.0 statistical software (IBM SPSS Statistics) and GraphPad Prism 8.0 software (GraphPad Software Inc.). The presentation of data conformed to mean ± standard deviation. Statistical significance in two groups was analyzed by the *t* test, and statistical significance in multiple groups was analyzed by one‐way or two‐way analysis of variance (ANOVA), followed by Tukey's multiple comparison test. *p* < .05 was regarded as statistical significance.

## 3 RESULTS

### 3.1 Inhibition of KLF9 alleviates COPD‐induced airway inflammation

**Figure 1:**
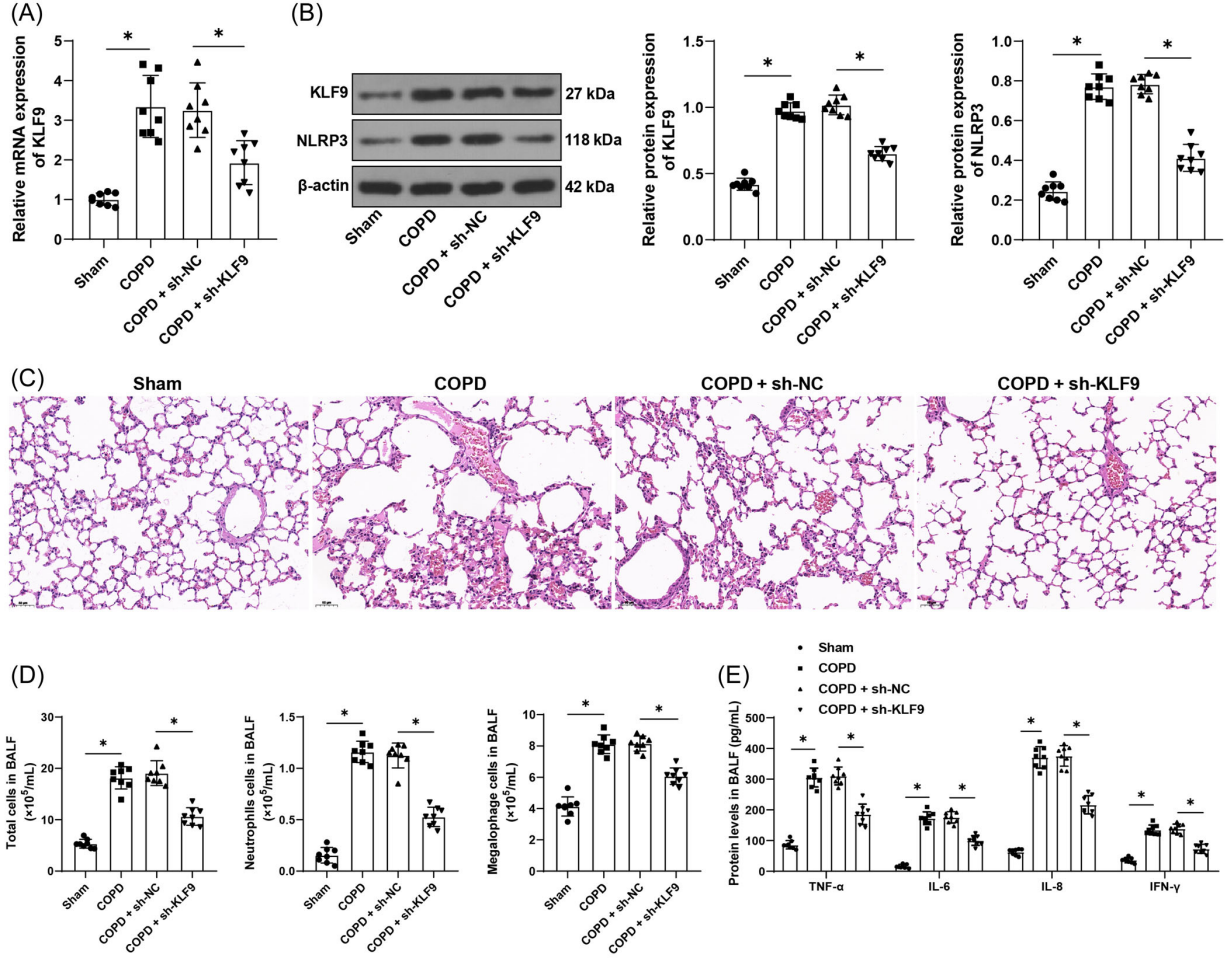
Inhibition of KLF9 alleviates COPD‐induced airway inflammation. Mice were injected with lentiviral vector (sh‐KLF9) via the caudal vein, with sh‐NC as the control. COPD mice were established through smoke exposure. (A) qRT‐PCR determined KLF9 RNA levels in lung tissues. (B) Expression levels of KLF9 and NLRP3 in lung tissues were determined by Western blot assay. (C) Pathological structure of lung tissue was observed by H&E staining, bar = 50 μm, magnification ×200. (D) The number of total cells, neutrophils, and macrophages in collected BALF. (E) Levels of TNF‐α, IL‐6, IL‐8, and IFN‐γ in BALF were determined by ELISA; *n* = 8, *p < .05. Data in (A), (B), and (D) were analyzed by one‐way ANOVA, and data in (E) were analyzed by two‐way ANOVA, followed by Tukey's multiple comparison test. ANOVA, analysis of variance; COPD, chronic obstructive pulmonary disease; ELISA, enzyme‐linked immunosorbent assay; H&E, hematoxylin and eosin; qRT‐PCR, quantitative real‐time polymerase chain reaction.

First, we established the COPD mouse model through smoke exposure and downregulated KLF9 expression in vivo by injection of lentivirus vector (*p* < .05, Figure 1A,B). Through H&E staining, COPD mice were found to have presence of evidently inflammatory responses in lung tissues, partial rupture of the alveolar wall, large amount of inflammatory cell infiltration, and notable alveolar hemorrhage (Figure 1C). Smoke exposure also brought about increases in the number of total cells, neutrophils, and macrophages in BALF (*p* < .05, Figure 1D), and levels of inflammatory factors (TNF‐α, IL‐6, IL‐8, and IFN‐γ) in BALF (*p* < .05, Figure 1E). Next, we determined the expression pattern of NLRP3 inflammasome in lung tissues and our results elicited high expression of NLRP3 in the lungs of COPD mice (*p* < .05, Figure 1B). The expression levels of KLF9 were upregulated in lung tissues after smoke exposure (*p* < .05, Figure 1A,B), and inhibition of KLF9 significantly alleviated airway inflammation of COPD mice (*p* < .05, Figure 1C–E) and reduced NLRP3 expression levels in lung tissues (*p* < .05, Figure 1B).

### 3.2 Inhibition of KLF9 alleviates CSE‐induced inflammation in vitro

**Figure 2:**
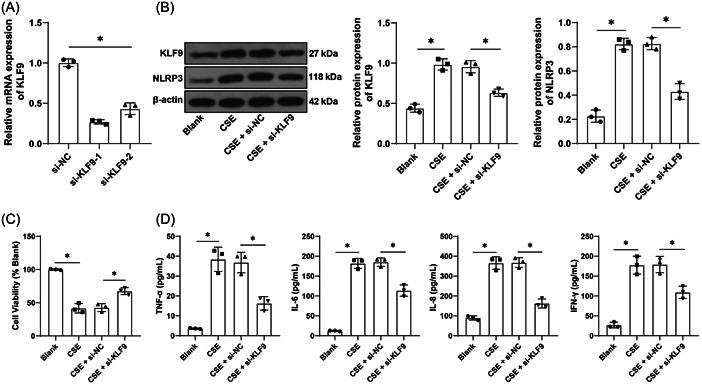
Inhibition of KLF9 alleviates CSE‐induced inflammation in vitro. BEAS‐2B cells were transfected with two siRNAs targeting KLF9 (si‐KLF9‐1 and si‐KLF9‐2), with si‐NC as the control. (A) Knockdown effectiveness was tested by qRT‐PCR; si‐KLF9‐1 with better knockdown effectiveness was selected for the subsequent assays and BEAS‐2B cells were treated with 5% CSE. (B) Protein levels of KLF9 and NLRP3 were determined by Western blot assay. (C) Cell viability was assessed by cell counting kit‐8 assay. (D) Contents of TNF‐α, IL‐6, IL‐8, and IFN‐γ were determined by ELISA. Cell experiments were performed three times independently, *p < .05. Data were analyzed by one‐way ANOVA, followed by Tukey's multiple comparison test. ANOVA, analysis of variance; CSE, cigarette smoke extract; ELISA, enzyme‐linked immunosorbent assay; qRT‐PCR, quantitative real‐time polymerase chain reaction.

Next, BEAS‐2B cells were cultured in vitro and later transfected with two strands of si‐KLF9, from which we selected si‐KLF9‐1 with better knockdown effectiveness for the subsequent assay (*p* < .05, Figure 2A). The COPD cell model was established in vitro using CSE and the protein levels of KLF9 were elevated by CSE treatment (*p* < .05, Figure 2B). After knockdown of KLF9, CSE‐induced inhibition of cell viability was reversed (*p* < .05, Figure 2C) and levels of TNF‐α, IL‐6, IL‐8, and IFN‐γ were significantly decreased (*p* < .05, Figure 2D), and NLRP3 expression was repressed (*p* < .05, Figure 2B). The above results suggested that inhibition of KLF9 could alleviate CSE‐induced inflammation in vitro.

### 3.3 KLF9 promotes miR‐494‐3p expression to repress PTEN expression

**Figure 3:**
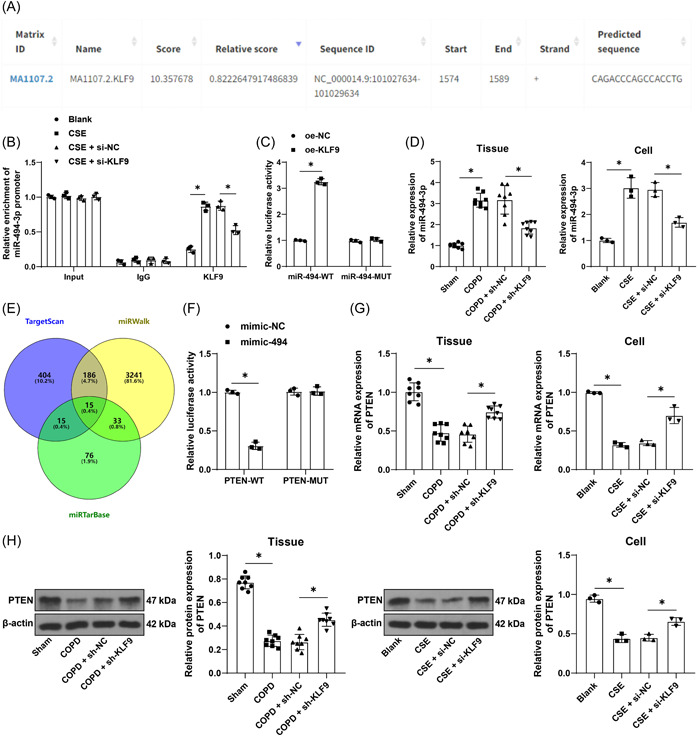
KLF9 promotes miR‐494‐3p expression to repress PTEN expression. (A) The binding site of KLF9 and the miR‐494‐3p promoter sequence was analyzed by the JASPAR database. (B) Enrichment of KLF9 in miR‐494‐3p promoter sequence was analyzed by the ChIP assay, with IgG as the control. (C) The binding of KLF9 to the miR‐494‐3p promoter sequence was verified by the dual‐luciferase assay. (D) Levels of miR‐494‐3p in tissues and cells were determined by qRT‐PCR. (E) The Venn plot showed the downstream target genes of miR‐494‐3p predicted by the TargetScan, miRWalk, and miRTarBase databases and intersections of genes. (F) The binding of miR‐494‐3p to PTEN 3′UTR sequence was testified by the dual‐luciferase assay. (G, H) PTEN expression levels in tissues and cells were determined by qRT‐PCR and Western blot assay. *n* = 8, cell experiments were performed three times independently, *p < .05. Data in (B), (C), and (F) were analyzed by two‐way ANOVA and data in (D), (G), and (H) were analyzed by one‐way ANOVA, followed by Tukey's multiple comparison test. ANOVA, analysis of variance; ChIP, chromatin immunoprecipitation; PTEN, phosphatase and tensin homolog; qRT‐PCR, quantitative real‐time polymerase chain reaction.

The JASPAR database predicted that KLF9 can bind to the miR‐494‐3p promoter sequence (Figure 3A). The ChIP assay revealed that KLF9 was abundantly enriched in the miR‐494‐3p promoter sequence and the enrichment was reduced by inhibition of KLF9 (*p* < .05, Figure 3B). The dual‐luciferase assay further verified that the binding of KLF9 to the miR‐494‐3p promoter sequence significantly increased luciferase activity (*p* < .05, Figure 3C). In addition, we determined miR‐494‐3p expression in tissues and cells, and the results showed that smoke exposure and CSE treatment elevated miR‐494‐3p expression in lung tissues and cells and inhibition of KLF9 significantly downregulated miR‐494‐3p expression (*p* < .05, Figure 3D). The downstream target genes of miR‐494‐3p were predicted by the TargetScan, miRWalk, and miRTarBase databases, and intersections of genes were identified (Figure 3E), among which the weak expression of PTEN in COPD has been previously documented.^27^, ^28^ The dual‐luciferase assay showed that the binding of miR‐494‐3p mimic to PTEN 3′UTR sequence inhibited luciferase activity (*p* < .05, Figure 3F). Additionally, the expression trend of PTEN in tissues and cells was opposite to that of miR‐494‐3p (*p* < .05, Figure 3G,H). These findings elicited that KLF9 promoted miR‐494‐3p expression to repress PTEN expression in COPD.

### 3.4 Upregulation of miR‐494‐3p reverses the alleviative role of KLF9 silencing in CSE‐induced inflammation

**Figure 4:**
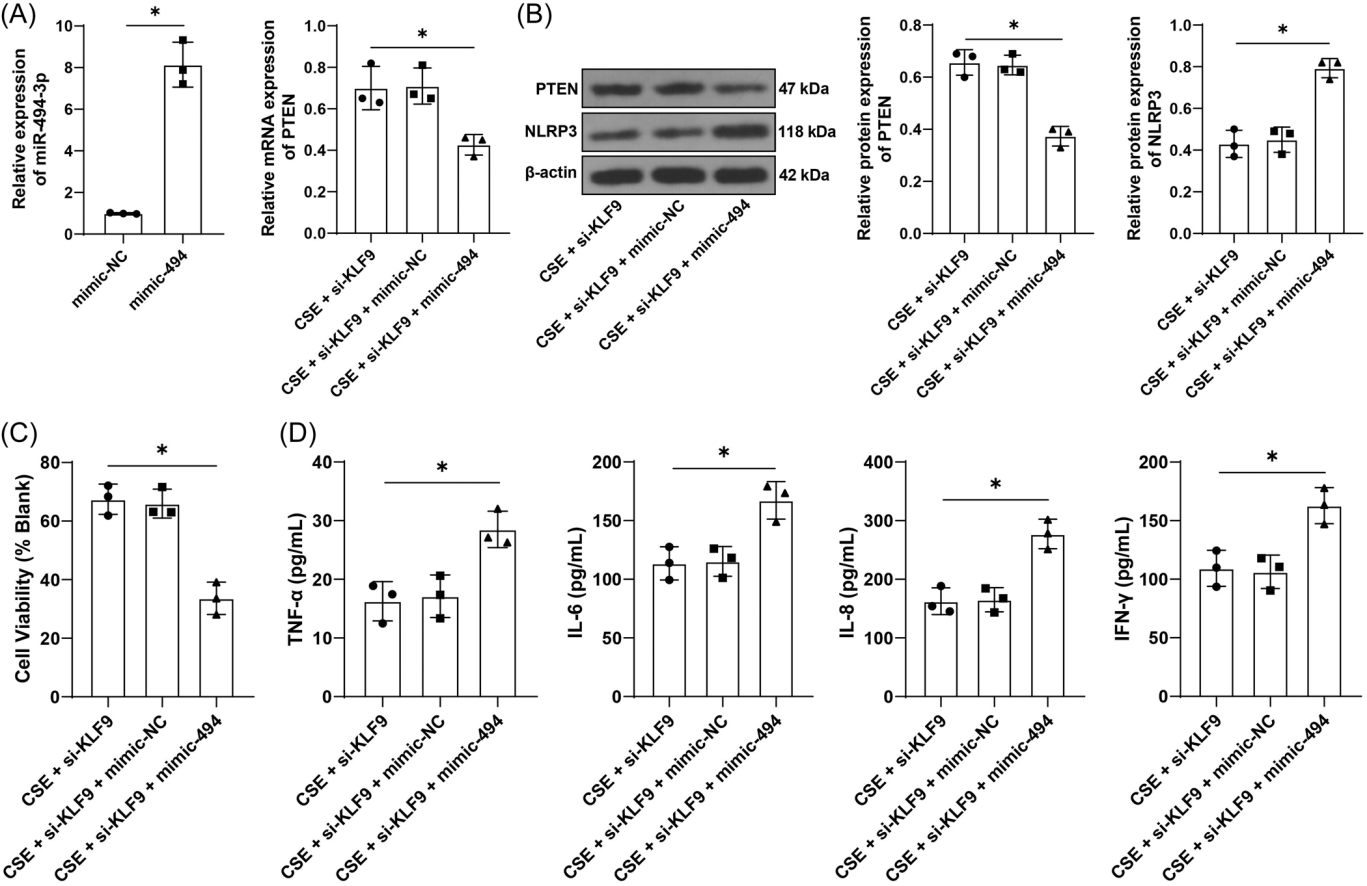
Upregulation of miR‐494‐3p reverses the alleviative role of KLF9 silencing in CSE‐induced inflammation. BEAS‐2B cells were transfected with miR‐494‐3p mimic (mimic‐494), with mimic‐NC as the control, followed by combined treatment with si‐KLF9 under the context of CSE treatment. (A) Levels of miR‐494‐3p and PTEN in cells were determined by qRT‐PCR. (B) Protein levels of PTEN and NLRP3 in cells were determined by Western blot assay. (C) Cell viability was assessed by the CCK‐8 assay. (D) Contents TNF‐α, IL‐6, IL‐8, and IFN‐γ in cells were determined by ELISA. Cell experiments were performed three times independently, *p < .05. Data in (A) (left) were analyzed by the *t* test, and data in (A) (right) and (B–D) were analyzed by one‐way ANOVA, followed by Tukey's multiple comparison test. ANOVA, analysis of variance; CCK‐8, cell counting kit‐8; CSE, cigarette smoke extract; ELISA, enzyme‐linked immunosorbent assay; qRT‐PCR, quantitative real‐time polymerase chain reaction.

To validate the above mechanism, BEAS‐2B cells were transfected with miR‐494‐3p mimic, resulting in upregulation of miR‐494‐3p (*p* < .05, Figure 4A), and then transfected cells were treated with si‐KLF9. Under CSE treatment, relative to inhibition of KLF9 alone, upregulation of miR‐494‐3p led to reductions in the expression levels of PTEN (*p* < .05, Figure 4A,B) and cell activity (*p* < .05, Figure 4C) while increases in levels of TNF‐α, IL‐6, IL‐8, and IFN‐γ (*p* < .05, Figure 4D) and NLRP3 expression (*p* < .05, Figure 4B). The above results indicated that upregulation of miR‐494‐3p reversed the alleviative role of KLF9 silencing in CSE‐induced inflammation.

### 3.5 Activation of NLRP3 reverses the alleviative role of KLF9 silencing in CSE‐induced inflammation

**Figure 5:**
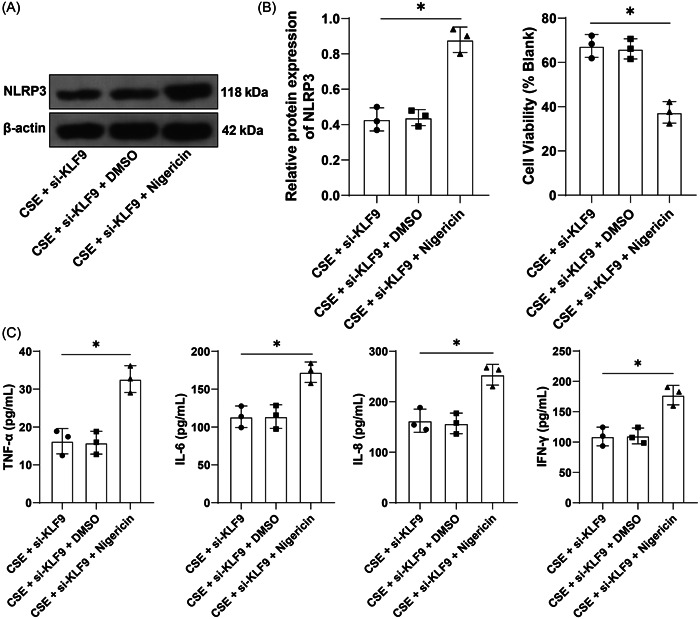
Activation of NLRP3 reverses the alleviative role of KLF9 silencing in CSE‐induced inflammation. NLRP3 agonist Nigericin was added into the culture medium at 10 µM concentration, with dimethylsulfoxide as the control, followed by combined treatment with si‐KLF9 under the context of CSE treatment. (A) Protein levels of NLRP3 were determined by western blot assay. (B) Cell viability was assessed by the CCK‐8 assay. (C) Contents TNF‐α, IL‐6, IL‐8, and IFN‐γ were determined by ELISA. Cell experiments were performed three times independently, *p < .05. Data were analyzed by one‐way ANOVA, followed by Tukey's multiple comparison test. ANOVA, analysis of variance; CCK‐8, cell counting kit‐8; CSE, cigarette smoke extract; ELISA, enzyme‐linked immunosorbent assay.

To verify whether the KLF9/miR‐494‐3p/PTEN axis mediates inflammation through NLRP3 inflammasome, BEAS‐2B cells were treated with a combination of NLRP3 agonist Nigericin and si‐KLF9. The results showed that relative to inhibition of KLF9 alone, Nigericin treatment increased NLRP3 expression (*p* < .05, Figure 5A), decreased cell viability (*p* < .05, Figure 5B), and increased the secretion of inflammatory factors (*p* < .05, Figure 5C). Altogether, activation of NLRP3 could reverse the alleviative role of KLF9 silencing in CSE‐induced inflammation.

## 4 DISCUSSION

COPD is an airway‐associated lung disorder that imposes great medical and socioeconomic burdens.^1^ Airway inflammation is closely implicated with the onset and exacerbation of COPD.^4^ With the advance in understanding of disease pathology and inflammatory phenotypes, molecular medicine targeting inflammation has become a promising approach to reverse the course of COPD.^4^ However, the currently identified inflammation‐sensitive molecules are questioned in application due to various drawbacks. For instance, the use of inhaled corticosteroids (ICS) can exert anti‐inflammatory effects in dual and triple therapies; however, it may present inconsistent therapeutical outcomes and cause side‐effects, such as osteoporosis and increased risk of infection^29^, ^30^; inhibition of cytokines (e.g., IL‐4, IL‐5, and IL‐13) seems feasible, but long‐term administration and high cost hinder their clinical application.^31^ In this regard, our study is of significance to increase the reservoir of molecules aiming for alleviation of airway inflammation in COPD.

KLF transcription factors are crucial regulators of inflammatory conditions, such as neutrophil/endothelial cell‐driven inflammation.^32^, ^33^ As a prominent member of the KLF family, KLF9 has been demonstrated to trigger lipopolysaccharide (LPS)‐induced pulmonary inflammation,^34^ TNF‐α‐induced synovial fibroblast inflammation,^35^ and high glucose‐induced trophoblast inflammation.^36^ Especially, KLF9 overexpression in the lungs of COPD mice and CSE‐treated BEAS‐2B cells has been identified in our study, which is consistent with a previous study reporting KLF9 elevation in the end‐stage lung of COPD.^10^ NLRP3 inflammasome is crucial for the initiation and amplification of airway inflammation and its activation is evidenced by upregulation of NLRP3, IL‐1β and caspase‐1.^6^ Our subsequent results elicited that inhibition of KLF9 reduces NLRP3 expression, inflammatory cell infiltration, and secretion of pro‐inflammatory TNF‐α, IL‐6, IL‐8, and IFN‐γ in BALF, which was consistent with in vitro results. Another investigation has documented that inhaled budesonide upregulates KLF9 in human airways,^37^ which may explicate that ICS also aggravate airway inflammation irrespective of its most likeness to benefit COPD patients.^38^ Collectively, the aforementioned findings suggested that inhibiting KLF9 may be a feasible target for alleviation of airway inflammation in COPD.

As a transcription factor, KLF9 is able to recognize the gene promoter to upregulate gene expression.^36^ However, when it comes to its interaction with miRNAs, most studies are illustrative of miRNAs‐mediated negative regulation of KLF9. A case in point is that miR‐494‐3p is the upstream gene that represses KLF9 expression in bladder cancer.^39^ By contrast, our study initially identified that miR‐494‐3p was a downstream target of KLF9 and KLF9 positively regulated miR‐494‐3p. Interestingly, miR‐494‐3p can be activated by oxidative stress in COPD cell model and its knockdown rescues cell senescence and inflammation.^17^ Furthermore, PTEN is a well‐established target for therapeutic intervention of COPD,^19^ and previous studies mainly discussed the interplay of PTEN with mitochondrial dysfunction, mitophagy, and senescence in COPD.^40^, ^41^ Less is known about its role in the inflammation state. Our data from databases and the dual‐luciferase assay verified the binding relationship between miR‐494‐3p and PTEN 3′UTR, which results in the repression of PTEN expression. Our following experimentation further suggested that miR‐494‐3p was upregulated while PTEN was downregulated in mice and cells after smoke exposure and CSE treatment, respectively, and overexpression treatment of miR‐494‐3p potentiated airway inflammation along with decreased PTEN expression and increased NLRP3 expression. Consistently, miR‐494‐3p regulates macrophage‐dependent immune and inflammatory responses with companionship of PTEN.^13^ Inhibition of PTEN is shown to increase pro‐inflammatory cytokine release and exacerbate airflow obstruction in COPD.^42^ However, another study has reported that miR‐494‐3p moderates acute lung injury‐induced inflammation by blocking NLRP3 activation.^43^ It seems plausible that although miR‐494‐3p is detrimental for airway inflammation, it can reduce the risk of incident pneumonia following COPD.

The activation of NLRP3 inflammasome in COPD can be attributed to noxious particles, such as cigarette smoke and PM2.5^5^ and such activation may be present in macrophages.^44^ NLRP3 inflammasome effectors, IL‐1β and IL‐18, are correlated with COPD‐like symptoms and the disease severity.^45^ Moreover, corticosteroids alleviate LPS‐induced inflammation and lung injury by blocking the activation of NLRP3 inflammasome,^46^ highlighting that NLRP3 inflammasome can be a drug target for COPD treatment and involves the pathogenesis of COPD, particularly those induced by infection. In our study, we observed that NLRP3 inflammasome was the effector of the KLF9/miR‐494‐3p/PTEN axis and activation of NLRP3 using nigericin reversed the alleviative role of silencing KLF9 in CSE‐induced inflammation, suggesting the involvement of the KLF9/miR‐494‐3p/PTEN axis in NLRP3 inflammasome‐mediated airway inflammation.

However, this study also has several limitations. First, since our study is the first to explore the role of KLF9 in COPD airway inflammation, we have not yet tested which specific cells in lung tissues have upregulated KLF9 expression but merely detected the overall KLF9 expression in lung tissues. In the future, we will conduct a detailed analysis of the expression of KLF9 in different cells, such as immune cells, endothelial cells, and epithelial cells, to comprehensively analyze the regulatory role of KLF9 in COPD. Second, bronchial epithelial cells perform some immune functions, but specialized immune cells such as macrophages make a greater contribution. Particularly, activation of macrophage NLRP3 inflammasome is known to play a crucial role in the chronic inflammation associated with COPD. Nevertheless, given the heterogeneity, multiple subpopulations, and different effector functions of pulmonary macrophages in different environments, we did not select macrophages for relevant experiments. Third, our mechanism analysis was mainly conducted at the cellular level, lacking in vivo validation. In addition, the COPD model was established by smoke exposure, which cannot fully represent the etiology of COPD. Future endeavors shall be granted to validate the mechanism in vivo and investigate whether KLF9 has the same regulatory mechanism in COPD caused by other etiologies.

## 5 CONCLUSIONS

To conclude, our study is the first to explore the role of KLF9 in smoke exposure‐induced COPD and its regulatory mechanism in airway inflammation. Our findings suggested that KLF9 is upregulated in COPD and its silencing alleviates NLRP3 inflammasome‐mediated airway inflammation through the miR‐494‐3p/PTEN axis, which proposed KLF9, miR‐494‐3p, PTEN as potential targets for the diagnosis and treatment of COPD.

## AUTHOR CONTRIBUTIONS

**Peijie Gu**: Conceptualization; formal analysis; writing—original draft; writing—review and editing. **Zhen Wang**: Writing—original draft. **Xin Yu**: Resources. **Nan Wu**: Investigation; writing—original draft. **Liang Wu**: Software. **Yihang Li**: Writing—original draft. **Xiaodong Hu**: Conceptualization; data curation; formal analysis; supervision; writing—original draft; writing—review and editing.

## CONFLICT OF INTEREST STATEMENT

The authors declare no conflict of interest.

## Data Availability

The data that support the findings of this study are available from the corresponding author upon reasonable request.
